# Measurement of Human Semicircular Canal Spatial Attitude

**DOI:** 10.3389/fneur.2021.741948

**Published:** 2021-09-24

**Authors:** Shuzhi Wu, Ping Lin, Yanyan Zheng, Yifei Zhou, Zhaobang Liu, Xiaokai Yang

**Affiliations:** ^1^Neurology Department, Third Affiliated Hospital of Shanghai University, Wenzhou Third Clinical Institute Affiliated to Wenzhou Medical University, Wenzhou People's Hospital, Wenzhou, China; ^2^ENT Department, Third Affiliated Hospital of Shanghai University, Wenzhou Third Clinical Institute Affiliated to Wenzhou Medical University, Wenzhou People's Hospital, Wenzhou, China; ^3^Department of Medical Imaging, Suzhou Institute of Biomedical Engineering and Technology, Chinese Academy of Sciences, Suzhou, China

**Keywords:** semicircular canal, measurement, spatial direction, magnetic resonance imaging, human, model

## Abstract

Located deep in the temporal bone, the semicircular canal is a subtle structure that requires a spatial coordinate system for measurement and observation. In this study, 55 semicircular canal and eyeball models were obtained by segmentation of MRI data. The spatial coordinate system was established by taking the top of the common crus and the bottom of the eyeball as the horizontal plane. First, the plane equation was established according to the centerline of the semicircular canals. Then, according to the parameters of the plane equation, the plane normal vectors were obtained. Finally, the average unit normal vector of each semicircular canal plane was obtained by calculating the average value of the vectors. The standard normal vectors of the and left posterior semicircular canal, superior semicircular canal and lateral semicircular canal were [−0.651, 0.702, 0.287], [0.749, 0.577, 0.324], [−0.017, −0.299, 0.954], [0.660, 0.702, 0.266], [−0.739, 0.588, 0.329], [0.025, −0.279, 0.960]. The different angles for the different ways of calculating the standard normal vectors of the right and left posterior semicircular canal, superior semicircular canal and lateral semicircular canal were 0.011, 0.028, 0.008, 0.011, 0.024, and 0.006 degrees. The technology for measuring the semicircular canal spatial attitudes in this study are reliable, and the measurement results can guide vestibular function examinations and help with guiding the diagnosis and treatment of BPPV.

## 1. Introduction

The position and direction of the semicircular canals in three-dimensional coordinate systems are very important for vestibular function examinations and BPPV diagnosis and treatment. Because the semicircular canal is located in the deep part of the temporal bone, its structure is complicated and delicate, which makes it difficult to observe and measure directly. Moreover, it is necessary to establish a spatial coordinate system to measure the spatial direction of the semicircular canal, which means few studies have done this. In addition, the reported data are often inconsistent (1–4), often lack a reliable spatial coordinate system and are limited to studies of the semicircular canal morphology and the relative positional relationships of the semicircular canal (5–11). In the past, the study of semicircular canal anatomy usually used cadavers, which were limited in regards to anatomical morphology, and the spatial posture of the semicircular canal was not fully studied. In recent years, with the development of modern medical imaging and computer technology, the planar fitting and spatial posture measurement of semicircular canals based on 3D reconstruction technology have become the research focus of semicircular canal anatomy and morphology. Bradshaw studied the spatial posture of the semicircular canal through semiautomatic segmentation of the semicircular canal, automatic extraction of the semicircular canal centerline, and calculation of the semicircular canal plane fitting equation. This method is more reliable than the manual measurements reported in most of the literature, but its major disadvantage is that the head-space coordinate system has not been established. Because the plane equations of each semicircular canal are not included in the standard spatial coordinate system, the application of the measurement results is limited (7). The author explains that the reason why the head space coordinate system was not established is that it is difficult to determine the landmark positions necessary for establishing the coordinate system, and the scanning range is insufficient, resulting in omission of the landmark position. Cortés-Domínguez present a new method to extract the geometrical parameters of the semicircular canals of the inner ear without subjectivity or operator variability. The approach is based on an algorithm that computes the geometric characteristics of the ducts through a skeletonization process based on a fictitious repulsive force (12).

The commonly used three-dimensional coordinate systems include the Frankfort coordinate system (4, 11) and Reid's coordinate system (3). Frankfort horizontal is the plane passing through bilateral portions and the left orbitale. Reid's plane is defined as the plane that passes through the center of each external auditory canal and the inferior margin of the two orbits (1, 13). It is more difficult to find bone markers on MRI images. Some studies have tried to use semicircular canals and eyeballs to establish a spatial coordinate system, and it is considered that the plane formed by the bifurcation of the common crus and the center of the eyeball is horizontal (14, 15). However, additional studies have shown that the plane formed by the bifurcation of the common crus and the bottom of the eyeball is parallel to the Frankfort plane (16). In previous studies, the angle between the plane of the semicircular canal and the coordinate planes was calculated first, and then the average value of each angle was calculated. However, the spatial direction of the semicircular canal is determined by the normal vector of the semicircular canal (17). The coordinate value of the unit vector of the semicircular canal plane normal vector is equal to the cosine value of the direction angle. According to the rules of vector calculation, the average value of normal vectors in the semicircular canal plane should be the average value of the cosine of the direction angle, not the average value of the direction angle.

In this study, first, the spatial coordinate system was established through the semicircular canal and eyeball, then the plane equation was fitted according to the centerline of the semicircular canal, and the normal vector was obtained. Finally, the spatial posture of the human semicircular canal was measured using the means of the vectors and the direction angles.

## 2. Materials and Methods

### 2.1. Clinical Data

Fifty-five patients were included in this study with a normal inner ear as determined by MRI during the period from January 2014 to December 2019, including 26 men and 29 women, aged 43 ± years, range 5–77 years. The inclusion criteria were as follows: (1) the semicircular canal was clearly displayed without artifacts; (2) the bottom of the eyeball was clearly visible without artifacts; and (3) a complete semicircular canal model could be obtained by image segmentation. The exclusion criteria were as follows: (1) the existence of local lesions may affect the anatomical structure of the semicircular canals and (2) the presence of any abnormal head structures.

### 2.2. Examination Methods

A Siemens 1.5 T superconducting magnetic resonance system and standard head coil were used for the inner ear examination. A 3D constructive interference steady-state sequence (3D-CISS) (TR: 6.0 ms, TE: 2.7 ms, FOV: 135 × 18 mm, matrix: 256 × 102, thickness: 0.7 mm) was applied. Image processing and modeling The original image data were exported from PACS and saved in DICOM format. The images were obtained by reading the catalog with 3D slicer version 4.10.2 software (18), and the 3D-CISS sequence was automatically exported and saved in NII format. The image spacing was 0.3515625 × 0.3515625 × 0.6999983 mm. The manual segmentation process of the semicircular canal is tedious. Because the thresholds of the semicircular canal and cochlea are different, it is difficult to segment the inner ear based on the threshold method. The Segment Editor module of 3D slicer includes a threshold segmentation function and provides a variety of automatic threshold searching methods. We found that the Otsu method can quickly obtain the best segmentation threshold to achieve semicircular canal semiautomatic segmentation, but the model surface is not smooth enough (19). To improve the segmentation result, the markers of the segmentation results are expanded and extracted into the voxel model and then transformed into the surface model by using the marching cubes function of the grayscale model maker, which makes the surface of the semicircular canal model smoother. Furthermore, we also realized the automatic segmentation of semicircular canals using a 3D UNET-based deep convolutional neural network. Manual confirmation and correction are always necessary. The boundary between the eyeball and the surrounding tissues is clear, and the threshold-based method can quickly segment the eyeball.

### 2.3. Analysis of the Space Attitude of the Semicircular Canal

#### 2.3.1. Obtaining the Centerline of Semicircular Canal

**Figure 1:**
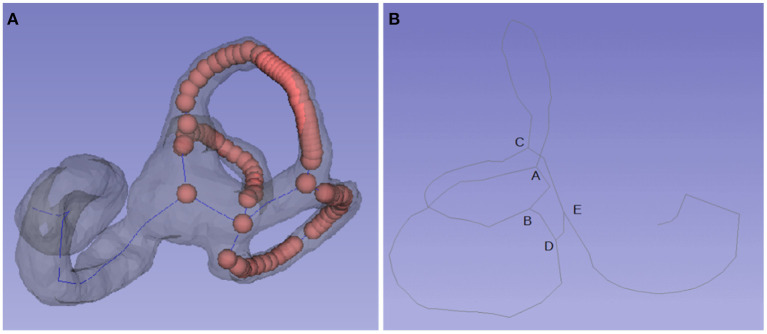
Centerline and key points of the semicircular canal. **(A)** The centerline of the semicircular canal is comprised of a set of points. **(B)** Most centerlines include four key points: A, B, C, and D. A is the bifurcation of the common crus, AD is the posterior semicircular canal, AC is the anterior semicircular canal, BC is the lateral semicircular canal, and point E is located in the utricle.

VMTK is an open-source c++ library using ITK and VTK for vascular structure segmentation, extraction, and analysis. It has a plug-in for 3D Slicer software. It mainly uses its vtkvmtkPolyDataCenterline function to obtain the inner ear centerline model (20). The centerline model of the inner ear is converted to a series of points (Figure 1). It can be seen from the analysis that the centerline of the whole inner ear includes three semicircular canal central rings. There are common intersections among the rings. The intersections appear at least three times in the array. The distances among the intersections of the semicircular canal central rings have a fixed pattern that can be used to exclude other abnormal intersections.

#### 2.3.2. Identification of the Semicircular Canal

The centerline of the inner ear can show its morphological characteristics. First, the intersection point and the line segments between the intersection points are analyzed. According to the length of the line segments, three semicircular canal central rings and a straight line from the cochlea to the utricle can be extracted. The length of the centerline of the posterior semicircular canal, the superior semicircular canal and the lateral semicircular canal were decreased in turn, but sometimes the connection from the cochlea to the utricle and other abnormal connections affected the judgment. Specific anatomical structures can be distinguished according to the relative spatial position and length of the lines. The midpoint of the central ring of the posterior semicircular canal is in the rearmost position, the midpoint of the central ring of the superior semicircular canal is in the uppermost position, the midpoint of the central ring of the lateral semicircular canal is in the outermost position, and the midpoint of the connecting line from the cochlea to the utricle is in the foremost position.

#### 2.3.3. Identify the Critical Intersections

The semicircular canal model obtained by semiautomatic segmentation is consistent in shape. Most of the central line of the semicircular canal includes four key points, including point A (the bifurcation of the common crus), which connects the posterior semicircular canal (AD) and the anterior semicircular canal (AC); point C, which connects the superior semicircular canal (CA), the lateral semicircular canal (CB) and the utricle (E); B and D, which may coincide; and point D, which connects the posterior semicircular canal (DA) and the utricle (DE) (Figure 1). The superior intersection of the posterior semicircular canal and the superior semicircular canal is at the top of the common corus (A), and then other key points D, C, B, and E can be identified in turn.

#### 2.3.4. Establishment of the Standard Spatial Coordinate System

**Figure 2:**
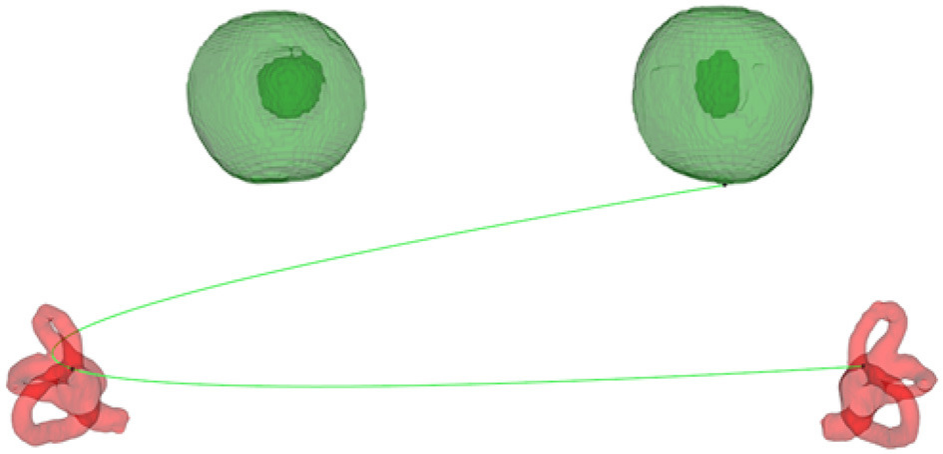
The plane formed by the bifurcation of the common crus and the bottom of the eyeball.

First, the fundus plane was formed by taking the lowest point of the eyeball (the minimum *Z*-value) and the bifurcation of the bilateral common crus, and the mathematical equation of the plane was calculated (Figure 2). Then, adjustment of the plane is needed. The distance from each point of the eyeball to the plane can be calculated by the mathematical equation of the plane with the coordinate values of the point. The point with the longest distance is taken as the lowest point of the eyeball in the standard coordinate system, which forms a horizontal plane with the bifurcation of the bilateral common crus. To establish the spatial coordinate system, the coordinate axes must be determined. The normal vector of the horizontal plane is the Z-axis. Because the semicircular canals are symmetrical left and right, the line connecting the bilateral crus bifurcation is selected as the X-axis, and the Y-axis is further determined as the cross product of the X-axis and Z-axis. To maintain consistency with the spatial coordinate system of the 3D slicer software, the right-hand Cartesian coordinate system is adopted.

#### 2.3.5. Fitting Equation for the Semicircular Canal Plane

**Figure 3:**
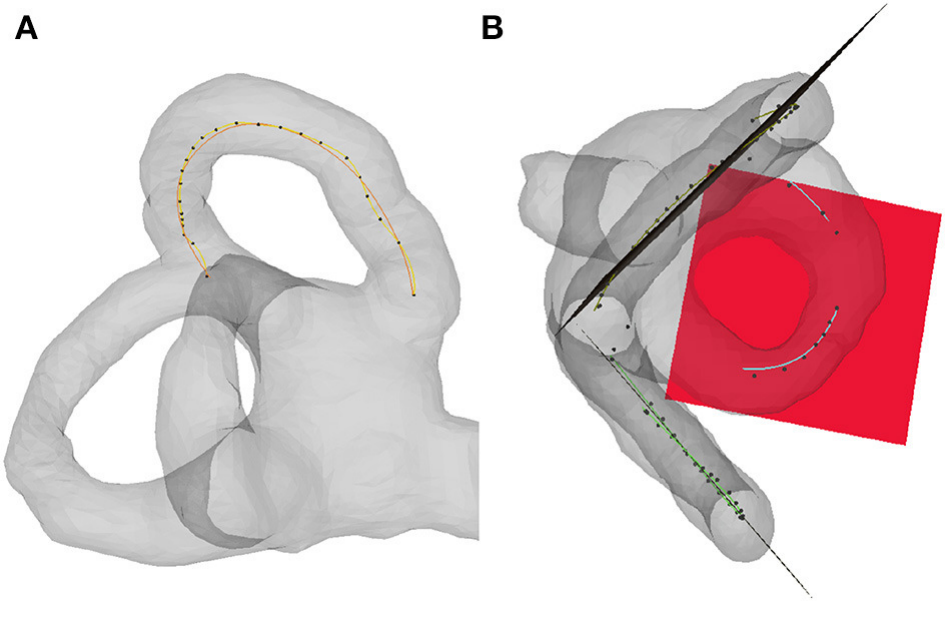
Semicircular canal plane fitting. **(A)** Fitting spline curve of the semicircular canal centerline **(B)** Fitting the plane of the semicircular canal according to the spline curve.

The centerline of the semicircular canal can be transformed into a group of points with different adjacent distances. The distribution of the points in the curved part is dense, while that in the straight part is sparse. It is inaccurate to fit the plane directly according to these points. To improve the fitting results, first, the spline curves were fitted according to the central line of the semicircular canal, and then the points of the spline curves were calculated according to the least square method to fit the semicircular canal plane (Figure 3). To determine its position in the standard spatial coordinate system, it is necessary to further calculate the angle (direction angle) between its normal vector and each axis in the standard spatial coordinate system and then establish the unit normal vector in the standard space coordinate system according to the cosine of the direction angle (direction cosine).

#### 2.3.6. Measurement of the Semicircular Canal Attitude

First, the plane equations of the posterior semicircular canal, the superior semicircular canal, and the lateral semicircular canal are calculated, and the unit vector of the normal vector in the standard spatial coordinate system is obtained. There are two ways to measure the spatial posture of the human semicircular canal, including the means of the vectors and the means of the direction angles. The average unit normal vector of each semicircular canal plane is obtained by calculating the sum of the normal vectors of the posterior semicircular canal plane, superior semicircular canal plane, and the lateral semicircular canal plane. The direction angle can be calculated by using the arccosine function. The deviation range of the unit normal vector is obtained by calculating the angle between each unit normal vector and the average unit normal vector. The traditional method is to calculate the angle between the semicircular canal plane and the coordinate plane and then to calculate the average direction angle. The standard normal vectors can be calculated using the cosine of the average direction angle.

#### 2.3.7. Display and Observation of the Normal Vector Distribution

The unit normal vector of each semicircular plane is distributed on a circle with a radius of 1. For convenience of observation, the radius is expanded to 100, the normal vectors of the different semicircular canal planes are marked with different color points, and the average normal vectors are represented by arrows.

## 3. Results

### 3.1. Segmentation for the Semicircular Canal

**Figure 4:**
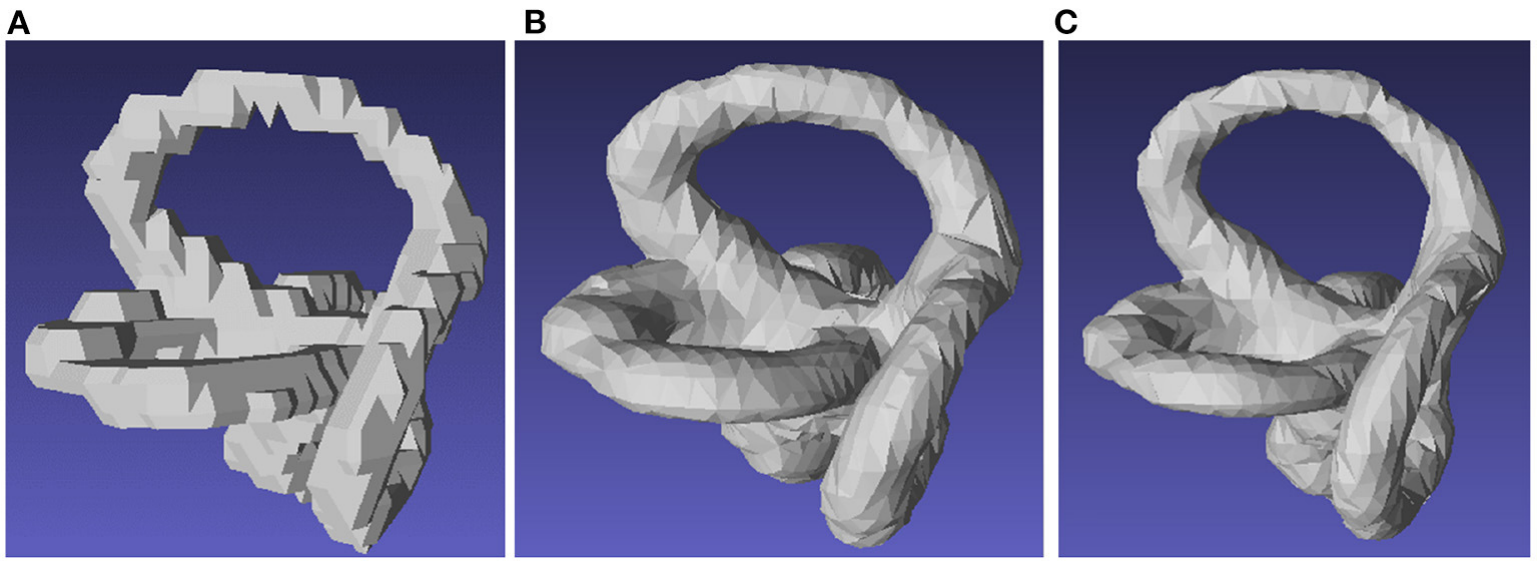
Segmentation of the semicircular canal. **(A)** Manual segmentation of the semicircular canal. **(B)** Segmentation of the semicircular canal using the marching cube function. **(C)** Segmentation of the semicircular canal using a 3D UNET-based deep convolutional neural network.

Seventy-one cases of semicircular canals and eyeballs were automatically segmented based on the deep learning method and exported to STL format. After manual confirmation and correction, 55 cases were included in this study (Figure 4).

### 3.2. Measurement of the Space Attitude of the Semicircular Canal

The spatial coordinate value of the vector is related to the head position and the selected spatial coordinate system. In our measurement environment, when the head looks forward, the x-axis is positive to the left, the y-axis is positive to the outside, and the z-axis is positive to the top.

The standard normal vectors of the right and left posterior semicircular canal, superior semicircular canal and the lateral semicircular canal were [−0.651, 0.702, 0.287], [0.749, 0.577, 0.324], [−0.017, −0.299, 0.954], [0.660, 0.702, 0.266], [−0.739, 0.588, 0.329], [0.025, −0.279, 0.960].

With the standard normal vectors, the angles between the semicircular canal plane and the corresponding sagittal plane, coronal plane, and horizontal plane can be calculated using the arccosine function (see Table 1).

**Table 1:** Calculate the angle between the plane of the semicircular canal and the coordinate plane by the vector method.

**Semicircular canal**	**Sagittal plane (**°**)**	**Coronal plane (**°**)**	**Horizontal plane (**°**)**	**Deviation range (x ± s**°**)**
RP	130.62	45.37	73.29	5.26 ± 2.52
RA	41.47	54.74	71.07	5.31 ± 2.69
RH	90.95	107.38	17.41	6.94 ± 3.64
LP	48.67	45.39	74.57	5.40 ± 2.92
LA	137.62	53.96	70.80	5.60 ± 2.98
LH	88.55	106.18	16.25	6.73 ± 3.15

*P, posterior semicircular canal; A, superior semicircular canal; H, lateral semicircular canal; R, right; L, left*.

**Figure 5:**
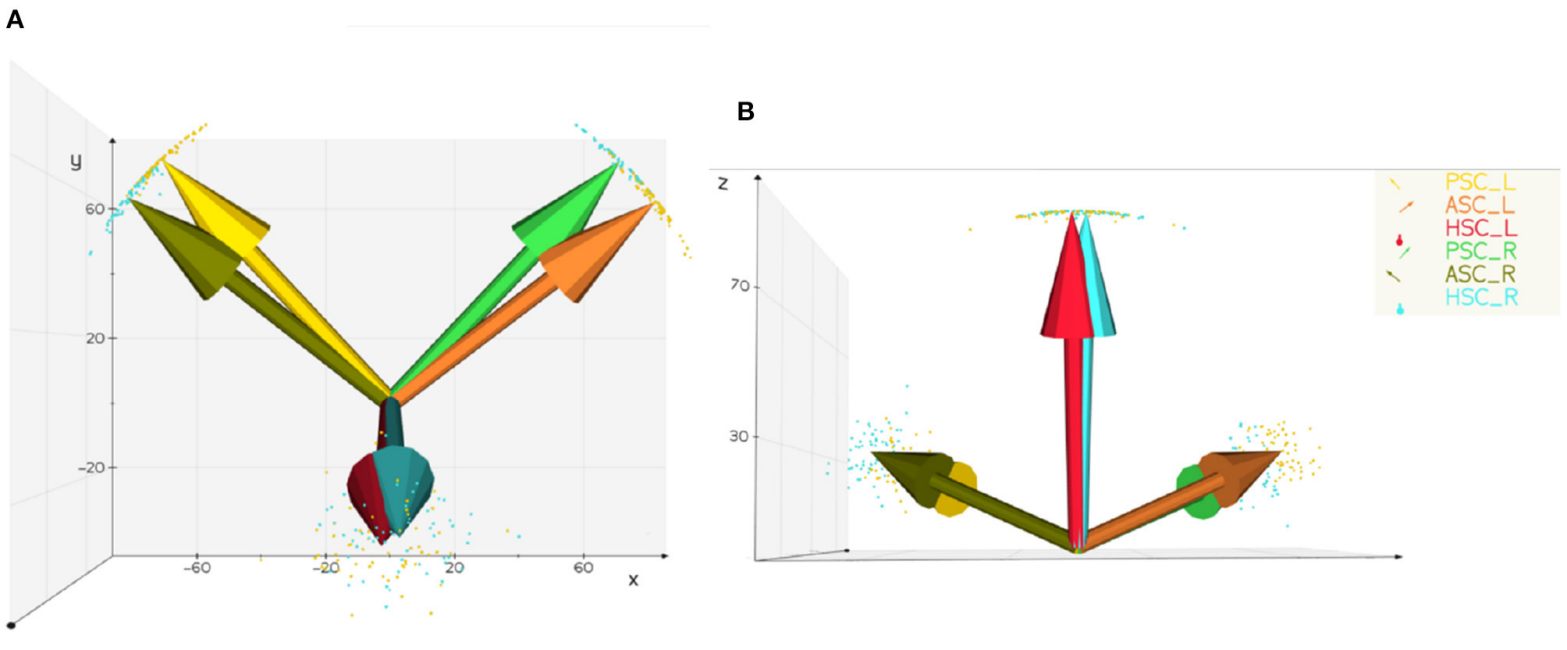
Distribution and average normal vector of the semicircular canal plane. PSC, posterior semicircular canal; ASC, superior semicircular canal; HSC, lateral semicircular canal; R, right; L, left. **(A)** Vertical view scatter points represent the unit normal vector and arrows represent the average normal vector **(B)** back view the plane normal vectors of the left and right lateral semicircular canals are nearly parallel, and the plane normal vectors of the posterior semicircular canals and the contralateral superior semicircular canals have only a small intersection angle.

The traditional method is to calculate the angle between the semicircular canal plane and the coordinate plane and then obtaining the average direction angle (see Table 2). In this way, the standard normal vectors of the right and left posterior semicircular canal, superior semicircular canal and lateral semicircular canal were [−0.651, 0.703, 0.287], [0.750, 0.577, 0.324], [−0.017, −0.299, 0.954], [0.660, 0.702, 0.266], [−0.739, 0.588, 0.329], [0.025, −0.279, 0.9602]. The different angles between the different ways of calculating the standard normal vectors of the right and left posterior semicircular canal, the superior semicircular canal and the lateral semicircular canal were 0.011, 0.028, 0.008, 0.011, 0.024, and 0.006 degrees. The distribution and relationships of the normal vectors of the semicircular canal plane can be observed directly (Figure 5).

**Table 2:** Calculate the angle between the plane of the semicircular canal and the coordinate plane by the average angle method.

**Semicircular canal**	**Sagittal plane (**°**)**	**Coronal plane (**°**)**	**Horizontal plane (**°**)**
RP	130.50 ± 4.12	45.50 ± 4.36	73.34 ± 3.97
RA	41.59 ± 4.83	54.84 ± 4.43	71.13 ± 3.63
RH	90.95 ± 5.92	107.28 ± 5.08	18.33 ± 5.20
LP	48.82 ± 4.21	45.57 ± 4.00	74.61 ± 4.61
LA	137.44 ± 4.59	54.12 ± 4.00	70.85 ± 4.75
LH	88.55 ± 5.89	106.09 ± 4.5	17.24 ± 4.57

*P, posterior semicircular canal; A, superior semicircular canal; H, lateral semicircular canal; R, right; L, left*.

## 4. Discussion

A lot of methods have been proposed in the literature for the computation of centerlines from 3d models (12). Skeletonize's skeletonize function supports 3D image skeleton extraction, but the 3D Slicer software has a VMTK plug-in, which can call its vtkvmtkPolyDataCenterline function in Python programming to obtain the centerline of the inner ear model and can observe and analyze it in the 3D Slicer software. The algorithm implemented in vmtk deals with the computation of centerlines starting from surface models, and has the advantage that it is well characterized mathematically and quite stable to perturbations on the surface.

**Figure 6:**
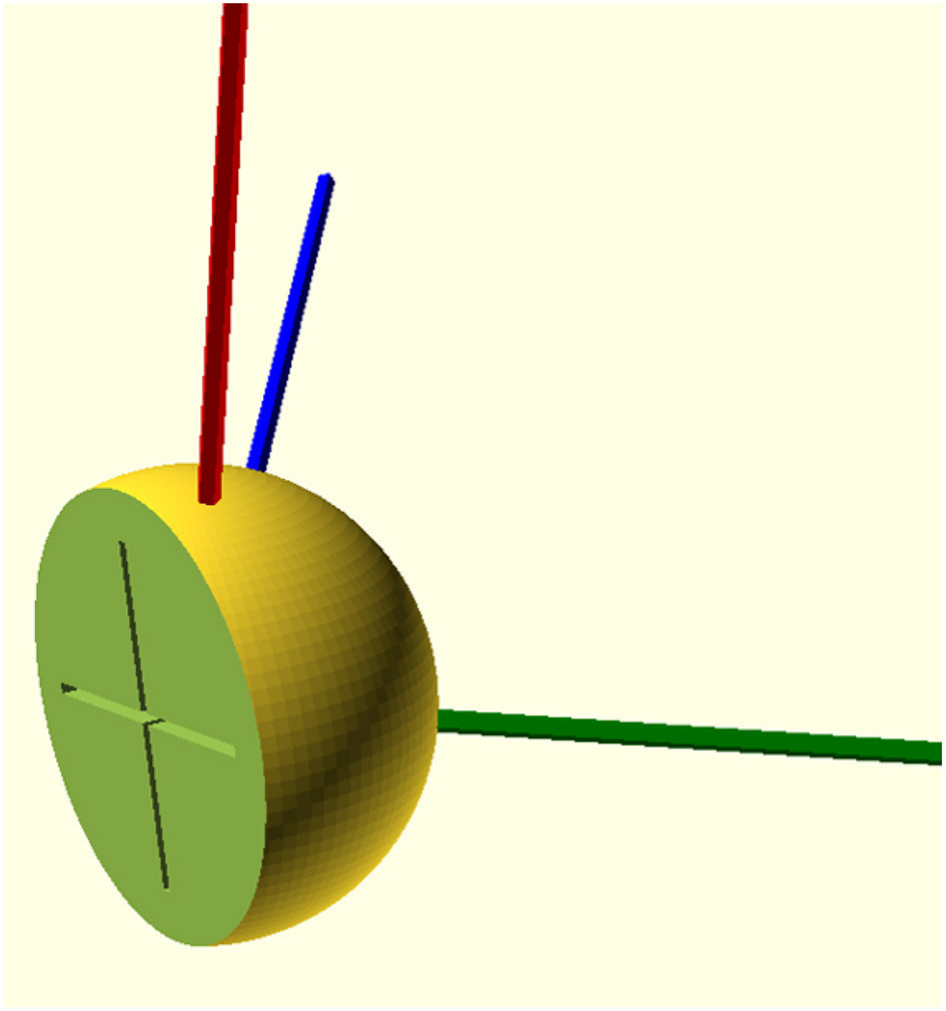
Solid model of the human semicircular canal spatial attitude. Red, blue, and green represent the normal vectors of the right lateral semicircular canal plane, posterior semicircular canal plane, and superior semicircular canal plane, respectively. The medial cross represents the Y-axis and Z-axis.

Traditionally, different studies measuring the spatial direction of the semicircular canal always calculated the angles between each semicircular canal and the sagittal plane, coronal plane, and the cross-sectional plane and then took the average value of the angles in each coordinate plane as the spatial direction of the semicircular canal (Table 2). In the past two decades, the field of direction statistics has attracted much attention, and we can confirm that the average angle is officially defined as the angle of the average vector (17). Although the calculation methods of the average normal vector and average angle method are completely different in mathematics, we have little concern about the difference between them. With our data, the different angles between the calculated standard normal vectors were very small, and the largest difference was no more than 0.3 degrees for the lateral semicircular canal. According to the calculation principle, it can be inferred that when the data distribution is more concentrated, the results of the vector averaging method and angular averaging method are similar; when the data distribution is more dispersed, the difference between the results of the vector averaging method and angular averaging method will increase. The understanding of the spatial direction of the semicircular canal is often based on an intuitive understanding of the angle between two straight lines in a two-dimensional plane. For example, if the angle between the posterior semicircular canal and the sagittal plane is 45°, it is considered that the posterior semicircular canal is parallel to the sagittal plane after rotating around the Z-axis by 45°. This problem can also be transformed into the rotation of the normal vector of the semicircular canal plane. According to the angle between the plane of the semicircular canal and the coordinate plane in Table 1, the unit vector of the plane normal vector can be constructed, and the coordinate value is the cosine value of the direction angle. If you need to rotate the posterior semicircular canal to make it parallel to the sagittal plane, you only need to rotate its plane normal vector (blue line) to make it parallel to the X-axis, that is, the sagittal normal vector (Figure 6). Most of the previous studies are based on manual point taking and measurements, which cannot meet the need to evaluate the spatial attitudes of the semicircular canals accurately. Therefore, we used automatic semicircular plane fitting technology, applying vector means instead of direction angle means to obtain the semicircular spatial orientation, and we measured the semicircular spatial attitude, which can guide vestibular function examinations, such as head shaking tests, and help in guiding the diagnosis and treatment of BPPV disease.

## 5. Preprint Availability

The original version of the manuscript has been deposited on the researchsquare.com preprint server (manuscript id: rs-146523). The manuscript is available at: [https://www.researchsquare.com/article/rs-146523/v1] (https://www.researchsquare.com/article/rs-146523/v1).

## Data Availability Statement

The original contributions presented in the study are included in the article/Supplementary Material, further inquiries can be directed to the corresponding author/s.

## Ethics Statement

The study group confirms that the design and data collection of this retrospective research was performed in accordance with the Helsinki protocol and standard of Good Clinical Practice. Informed consent was not required because this was a retrospective study. All data were anonymized and deidentified before analyses. The study protocol was approved by the Ethics Committee of Wenzhou People's Hospital. The Ethics Committee determined that the study was exempt from an informed consent requirement since it was a review of existing clinical data with patient identifiers removed.

## Author Contributions

SW and XY conceived and designed the experiment and wrote the paper. YZhe, ZL, YZho, and PL conducted the experiment. All authors read and approved the manuscript.

## Funding

This study was funded by Wenzhou Municipal Science and Technology Bureau [Grant Nos. ZS2017020, Y2020420], Natural Science Foundation of Zhejiang Province [Grant No. LSY19H090002], and Foundation of Zhejiang Provincial Science and Technology Bureau [Grant No. LGF18H090007].

## Conflict of Interest

The authors declare that the research was conducted in the absence of any commercial or financial relationships that could be construed as a potential conflict of interest.

## Publisher's Note

All claims expressed in this article are solely those of the authors and do not necessarily represent those of their affiliated organizations, or those of the publisher, the editors and the reviewers. Any product that may be evaluated in this article, or claim that may be made by its manufacturer, is not guaranteed or endorsed by the publisher.
